# Using Mobile Phones to Examine and Enhance Perceptions of Control in Mildly Depressed and Nondepressed Volunteers: Intervention Study

**DOI:** 10.2196/10114

**Published:** 2018-11-09

**Authors:** Rachel Msetfi, Donal O'Sullivan, Amy Walsh, John Nelson, Pepijn Van de Ven

**Affiliations:** 1 Department of Psychology Health Research Institute University of Limerick Limerick Ireland; 2 Department of Electronics and Computer Engineering University of Limerick Limerick Ireland; 3 Department of Psychology University of Limerick Limerick Ireland

**Keywords:** perception of control, illusory control, well-being, depression, health, intervention, causal learning

## Abstract

**Background:**

Perceived control is strongly linked to healthy outcomes, mental healthiness, and psychological well-being. This is particularly important when people have little control over things that are happening to them. Perceived control studies have been performed extensively in laboratory settings and show that perceived control can be increased by experimental manipulations. Although these studies suggest that it may be possible to improve people’s mental health by increasing their perceived control, there is very little evidence to date to suggest that perceived control can also be influenced in the real world.

**Objective:**

The first aim of this study was to test for evidence of a link between noncontrol situations and psychological well-being in the real world using a mobile phone app. The second and arguably more important aim of the study was to test whether a simple instructional intervention on the nature of alternative causes would enhance people’s perceptions of their own control in these noncontrol situations.

**Methods:**

We implemented a behavioral action-outcome contingency judgment task using a mobile phone app. An opportunity sample of 106 healthy volunteers scoring low (n=56, no depression) or high (n=50, mild depression) on a depression scale participated. They were given no control over the occurrence of a low- or high-frequency stimulus that was embedded in everyday phone interactions during a typical day lasting 8 hours. The intervention involved instructions that either described a consistent alternative cause against which to assess their own control, or dynamic alternative causes of the outcome. Throughout the day, participants rated their own control over the stimulus using a quantitative judgment scale.

**Results:**

Participants with no evidence of depression overestimated their control, whereas those who were most depressed were more accurate in their control ratings. Instructions given to all participants about the nature of alternative causes significantly affected the pattern of perceived control ratings. Instructions describing discrete alternative causes enhanced perceived control for all participants, whereas dynamic alternative causes were linked to less perceived control.

**Conclusions:**

Perceptions of external causes are important to perceived control and can be used to enhance people’s perceptions. Theoretically motivated interventions can be used to enhance perceived control using mobile phone apps. This is the first study to do so in a real-world setting.

## Introduction

### Background

Perceived control is critical to health outcomes, mental healthiness, and psychological well-being. For example, numerous studies have measured perceived personal control using psychometric questionnaire measures and have shown direct relationships to health outcomes (eg, cancer [1], diabetes [2], heart disease [3], and treatment adherence and effectiveness [4]), with control mediating the negative consequences of adverse conditions [5]. A key idea is that when healthy people have no control over events, they tend towards an “illusory” perception of control (eg, [6]). This is thought of as a protective bias that supports people’s sense of control, and therefore well-being, when they cannot control things that are happening to them (eg, [7]). Conversely, people with depression are held to recognize situations in which they have no control all too well [8]. This “depressive realism” phenomenon may represent the absence of a healthy protective mechanism, with illusory control being an ingredient for positive physical and mental health [9,10]. Given the importance of perceived control for health, the aim of the current study was to assess for evidence of this phenomenon outside of the laboratory using a mobile phone app and to test whether a simple theoretically motivated intervention could enhance people’s perceptions of control in a healthy manner.

### Previous Research

Despite its importance, perceived control research suffers from a lack of studies carried out in real-world or applied settings. So far, laboratory-based research has been the only method of showing whether a person perceives that they have control when there is none. This is because the actual control a person has over a situation needs to be known and adjustable by the experimenter, and an accurate, objective measure of people’s experiences is required [11], which is almost never the case in the real world. Some methodologies used in this domain, for example, comparisons between self and observer ratings of a situation [12] or between personal and population risk (eg, of a cancer diagnosis [13]), have provided useful insight but cannot allow a definitive diagnosis of illusory perceived control.

**Figure 1:**
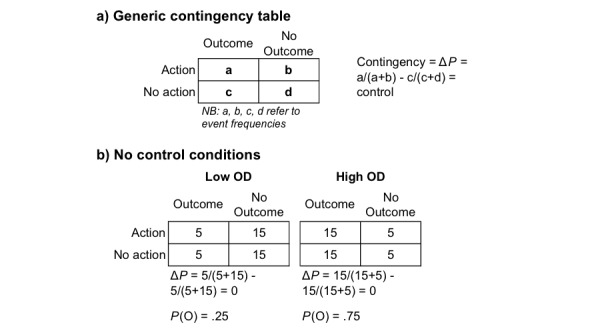
Generic (a) and specific (b) contingency relationships between the occurrence of an action and the occurrence of an outcome in conditions (b) in which there is "no control’"over the outcome. NB. The term "outcome density" (OD) refers to the probability of an outcome occurring over all events, P(O).

An objective measure of available control is clearly present in laboratory tasks involving “contingency judgments” as participants are exposed to carefully measured contingencies between their actions and outcomes [11]. The contingencies between a person’s actions and subsequent outcomes are defined using four event-outcome frequencies (Figure 1):

- a user action is followed by an outcome
- a user action is not followed by an outcome
- no action by the user is followed by an outcome
- no action by the user is not followed by an outcome (ie, no actions and no outcomes)

These events are usually programmed to occur over a short period of time and are quantified using the normative delta *P* (Δ*P*) metric [14]. Delta *P* is the difference between *P* (O|A), the probability of a user action (A) being followed by an outcome (O) and *P* (O|~A), the probability of the same outcome occurring when the user does not perform the action. Positive and negative Δ*P* values indicate the user has a certain control over the outcome, though in the case of a negative Δ*P* the outcome will be more likely to occur when there is no action by the user. A Δ*P* value equal to zero indicates the user cannot control the outcome through the action. In both of the specific examples given in Figure 1, the person has no control over salient outcomes (Δ*P*=0) but the frequency or density of outcomes varies from low to high (ie, low outcome density, high outcome density). Therefore, if accurate, people’s perceptions of control should not differ between these two conditions. However, numerous studies have shown that healthy people exposed to a high outcome density condition tend to overestimate their control relative to the low outcome density condition (eg, [15]) and relative to people who are depressed (eg, [8,16]). These findings have provided evidence for the link between illusory control and healthy states.

However, the requirement for careful experimental control means that the basic experimental findings have never been tested outside the laboratory. This raises key methodological concerns around external and ecological validity, of generalizability from one very specific control situation to the whole of life [17] and the difference between behavior instructed in the laboratory and that occurring naturally in the real world [18]. Such basic methodological critiques of perceived control research are well acknowledged [19] and have limited the potential for this area of research to result in interventions for applied settings, although laboratory-based interventions have begun to be tested [20].

Where laboratory research has been helpful is in shedding light on our understanding of the psychological processes underpinning perceived control and, theoretically, the factors that will enhance perceptions if used to formulate interventions. So, for example, we know that the perception of alternative causes of outcomes is a key moderator of perceived control [14,21]. Whether a rule-based normative model [14] or a process-based associative model [21] is preferred, one’s own control is evaluated against the control exerted by alternative causes. Other potential controlling causes are numerous, both inside and outside the laboratory, and include the environment or context in which events occur. For example, if a person wanted to control the heat level in a room using heating controls (action), an important alternative cause of heat variation would be the room itself and the effectiveness of the central heating system therein. In other words, the context is a key conditionalizer of control experience [22] and has been indicated as a key factor that discriminates healthy and mildly depressed people in their control perceptions [16]. These findings should theoretically [21] lend themselves to interventions that will influence people’s perceptions of alternative causes and enhance their feelings of control [20].

### Study Goals

Given the ubiquity of mobile phones in everyday life, and their potential as data gathering and intervention devices, the goals of this study were to investigate perceived control in the participant’s normal everyday environment and to test potential interventions that may increase a participant’s perceived control, whether or not they are depressed.

**Figure 2:**
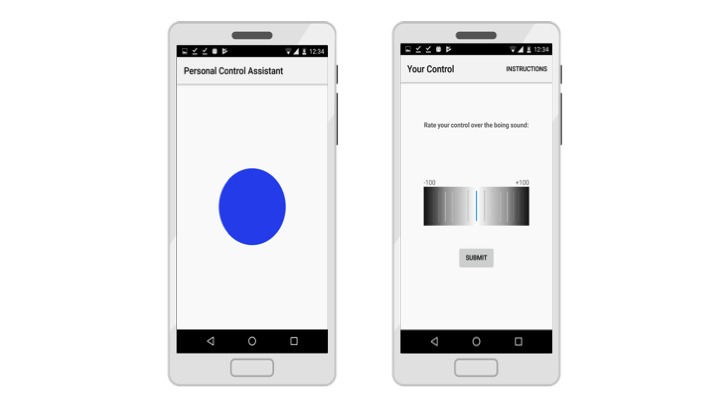
Contingency judgment task implemented as user-phone interactions, including action and auditory outcome stimulus, with control ratings given after every block of 8 interactions (trials).

To this end, we implemented a contingency judgment control task designed to run on Android mobile phones using the same conditions displayed in Figure 1. In order to maximize ecological validity, we used two strategies. First, experimental trials were embedded into participants’ everyday lives through user-phone interactions programmed to take place throughout the activities a participant would experience during a typical day (see Figure 2). Second, the user-phone interactions were modeled on very typical activities, such as when the phone user is alerted to the availability of new information (eg, a message), which is followed up by an action (eg, click to access) and an outcome (eg, message, picture, video). Thus, in our procedure, each trial was prompted by a standard Android alert message and consisted of a user action followed by the occurrence of a brief auditory stimulus or no auditory stimulus at the programmed probability. We also asked participants to rate their control over the auditory outcome at five time points throughout the day. Ratings were performed by scrolling a wheel to a value between -100 and 100, with -100 indicating a perception of complete preventative control and +100 indicating complete generative control. Note that such ratings can be mapped onto the programmed Δ*P* metric.

In addition, we programmed an intervention by manipulating the instructions given to participants about the nature of alternative causes of the auditory stimulus. We defined the alternative cause as a discrete, static entity constantly present throughout the task (ie, the mobile phone network) or as a dynamic entity that changed throughout the day (ie, the different places they visited during the day). This is important because, theoretically, there is a finite amount of causal control available to any given outcome [14,21]. This means that if one cause is seen as a strong “controller,” it is at the expense of all other potential causes, which will be seen as weak “controllers” of the outcome. Any cause that is constantly present (discrete context cause), especially when the outcome is absent, will be seen as a very weak controller, with other causes seeing their control enhanced. Therefore, we predict that participants in the discrete condition will see their actions as strong controllers of the outcome, and the extent of this control will be simply linked to the frequency with which the outcome occurs. The latter will increase the difference in control judgments between the low and high frequency conditions. Conversely, dynamic and therefore multiple alternative causes will have the opposite effect. We predict that people will learn as much about each cause as they do about the action. With causal control shared between so many potential causes, all of them will be perceived as weak, and the illusory control will be reduced (see also [16]).

To date, mobile devices have been used as data gatherers and to deliver therapy and interventions for numerous conditions (eg, depression [23]). Here, we also study the effects of levels of depressed mood because even minor elevations, so called mild depression [8], dysphoria [16], or even scores above the median on a depression scale [24], can have a significant impact on control perceptions. In this study, in order to test a random sample of the population and examine the effects of depression levels, we use the median as the cut-off score on the depression scale. Importantly, although mobile devices have been used very successfully to support healthy behavior change (eg, exercise [25]) and to promote healthy eating [26], they have never been used to assess or promote a general sense of control. Our study will be the first to do this.

## Methods

### Recruitment

Participants consisted of 106 university students who volunteered to participate by responding to an email advertisement and fulfilled the inclusion criteria: (1) access to an Android mobile phone and (2) over the age of 17. Volunteers completed the Beck Depression Inventory (BDI) [27] during a visit to the laboratory after which they were supervised in downloading and installing the mobile app.

On the basis of their BDI scores, participants were categorized as members of the low BDI group (BDI ≤5, n=56, female n=27, representing non-depression) or high BDI group (BDI >5, n=50, female n=36, representing mild depression). This cut-off value for group categorization has been used in other similar studies examining the effects of mild depression or dysphoria on contingency learning and represents the median BDI score in most samples [24,28]. Throughout this paper, we refer to high BDI groups or mild depression in order to describe and explain our findings.

Table 1 shows that the BDI groups produced significantly higher scores on other depression relevant scales, including anxiety and stress, specifically the Depression Anxiety Stress Scales (DASS). Given nonrandom assignment to BDI groups, we attempted to match these BDI groups on three characteristics relevant to performance on cognitive tasks and which are affected by levels of depression: age, IQ, and short-term memory capacity (see Measures section). We checked for any statistical differences between groups on these three measures. Thus, the groups were matched on age and estimated IQ scores [29] but not short-term memory capacity, as measured by digit span scores [30] (Table 1).

### Design

This study used a mixed 2×2×2×(5) factorial design, in which the between-groups variables were BDI group (2: low BDI, high BDI), outcome density (2: low, high), and alternative cause instructions (discrete, dynamic). The repeated measures variable was judgment block (5: 1-5). Participants made ratings of the control their actions had over the occurrence of the outcome. These were made using a +100 (complete control) through 0 (no control) to -100 (preventative control) scale, presented to the participant as a wheel (Figure 2). In addition, we recorded the number of actions made by each participant and calculated the contingency (actual Δ*P*) and outcome density experienced (based on the number and types of trials experienced), and the number of trials missed.

**Table 1:** Participant characteristics for each BDI group.

Measure	Low BDI^a^ (n=56)		High BDI (n=50)		Multivariate analysis of variance^b^	
	Mean	SE	Mean	SE	*F*	*P* value
Age	22.98	0.98	25.68	1.03	3.60	.06
Digit span score	7.61	0.18	6.90	0.19	7.47	.01
Estimated IQ	113.00	0.83	112.33	0.88	0.30	.58
BDI	2.30	0.65	12.56	0.68	119.21	<.001
DASS^c^-Anxiety	1.02	0.33	3.62	0.35	29.40	<.001
DASS-Depression	1.05	0.41	4.96	0.43	43.01	<.001
DASS-Stress	2.29	0.49	6.50	0.52	34.95	<.001

^a^BDI: Beck Depression Inventory.

^b^*df*=1, 104.

^c^DASS: Depression Anxiety Stress Scales.

### Measures

Participants completed the BDI; the DASS [31]; the digit span test, which provides a measure of short-term memory capacity; an estimated measure of pre-morbid IQ; and a number of other demographic items. Briefly, the digit span test requires participants to retain and repeat a randomized series of digits read to them at a rate of one per second. For the purposes of this study, the test was computerized. IQ was estimated using a formula applied to demographic variables. These measures are described in detail elsewhere [32]. In addition, participants completed a perception of control task, that was administered using a mobile phone.

### Perception of Control Task

The task was implemented using an app developed using an Android library for the context-aware delivery of messages to users’ mobile devices [33]. The app required wireless or network connectivity with the server initially in order for randomization to groups to take place and the experimental condition settings to be downloaded to the phone. Once this was complete, the app functioned independently and did not require continuous wireless or network connectivity. Incremental data upload to the server was programmed to take place as soon as connectivity was available, both during the task and once the task was finished.

The task was implemented as a discrete trials contingency judgment task, including 40 experimental trials lasting 6 seconds each, divided by intertrial intervals lasting on average 12 minutes (calculated using the Fleshler-Hoffman progression [34]). Each trial was prompted by a standard Android “notification” message (similar to those delivered to alert a user that a text message has arrived and is waiting to be read) that included a brief auditory and visual signal. A time limit of 2 minutes was given for participants to access the alert, after which the trial would be categorized as a “miss.” Under these circumstances, the alert would be removed from the screen and the next intertrial interval would commence. This procedure was used to ensure that the procedure lasted for the same duration for all participants. If the participant accessed the alert within the 2-minute time frame, the onscreen button would appear on the screen in 2 seconds. They would then have the opportunity to press a touch-screen button for 3 seconds. Whether or not the button was pressed within 3 seconds, an auditory outcome would sound for 1 second (outcome present), or it would not sound (outcome absent), depending on the programmed probability. Following each of these experimental trials, the intertrial interval would commence. Action-outcome contingencies were programmed as in Figure 1. Participants were either randomized to the low outcome density group (*P* [O|A]=.25, *P* [O|A]=.25) or the high outcome density group (*P* [O|A]=.75, *P* [O|A]=.75). Therefore, for all participants, the programmed contingency was zero, and they had no control over the sound’s occurrence. Every 8 trials, the participant would be asked to rate their own control using the previously described wheel (Figure 2). The procedure was programmed to last for approximately 8 hours and function during the participants’ typical day, in a similar manner to typical mobile phone interactions.

### Procedure

After having been fully briefed on arrival at the lab and having given informed consent, participants were asked for their demographic details, completed a series of questionnaires about their mood, and performed the digit span test. Following this, the experimenter helped participants download the app onto their own mobile phone and guided them through installing and activating the app. Once the app was activated, the participant was prompted to enter a code that would act as a unique identifier to allow matching of lab- and app-generated data. Following this, the instructions (Multimedia Appendix 1) were presented and participants told that they would interact with the phone during the course of the day, which would be opportunities to test if their button pressing controlled the sound occurrence. Following each block of 8 trials and the corresponding control rating, participants received an intervention message to prompt the participant to consider the influence of their context on their control. In the “discrete” condition, participants were told to think about the “control external factors have...this could be factors related to the phone, the mobile network or anything apart from your actions.” In the dynamic context condition, participants were asked to think about “control the place you are located in has...It’s important to note that you will change your location throughout the day. The place you are in could affect whether the boing sound occurs, regardless of your actions.” When the procedure was complete, the app provided debriefing information and links to support information provided on a webpage.

### Power

We conducted a priori power analyses, which indicated that a sample of 152 was required for a power of 0.8 to detect medium-sized between-group effects and a sample of 24 and 48 to detect repeated measures effects and interactions respectively. However, only 106 volunteers kept appointments at the laboratory to participate in the study. Based on the achieved sample size, compromise power to detect the repeated measures effects and interactions, which were the focus of our theoretical predictions, was high (>.99). The power to detect main effects of between-group variables was somewhat lower than we planned (0.70). In spite of this, the size of the key main effects was within the 90% confidence limits (BDI) leading us to conclude that this study provided an adequately powered test of the hypotheses.

Due to participants’ completing the task at the same time as their everyday activities, we anticipated missing data, in terms of trials and ratings, as well as issues such as loss of mobile phone battery. In this dataset, 10.6% of judgment values were missing. We therefore carried out multiple imputation, which involves replacing missing data with values generated from a series of multiple regression analyses including standard error and available parameter estimates. The fifth and most conservative iteration was used for the analyses reported in this paper.

### Perception of Control Task Validation

As this is the first time a contingency task has been tested outside the laboratory, it was important to track all user-phone interactions and report whether experience was in line with what we had programmed. On average, participants missed 11.4 trials (SE 0.79) of the programmed 40 and, as instructed, pressed the button on around half of the trials they engaged with (press proportion mean 0.58, SE 0.019). The actual contingency (Δ*P*) experienced was again close to 0 as programmed (mean 0.04, SE 0.02). Participants in the low outcome density condition experienced outcomes on an average of 11.7 trials out of 40 (29.2%, SE 2.7%) whereas participants in the high outcome density condition experienced outcomes on 27.7 trials out of 40 (69.2%, SE 2.5%). Overall, the recorded engagement with experimental trials and contingency experience was as we programmed.

## Results

In order to test the hypotheses, that (1) illusory control and depressive realism effects would be present, and (2) that the intervention would enhance ratings of control, a mixed factorial analysis of variance (Multimedia Appendix 2), which included actual Δ*P* experience as a covariate, was carried out on judgments of control. The alpha level was held at .05 throughout all analyses unless stated otherwise.

### Illusory Control and Depression Effects

There are two tests to demonstrate the presence or absence of an illusory control effect. The simplest and first test assumes that judgments that are different to zero represent illusory control. In this test, the higher the absolute number of the rating, the stronger the illusion of control. The second and more rigorous test assumes that people’s subjective perception of control scale might differ from the numeric scale on which they are asked to rate it and that positioning of ratings on the judgment scale is rather arbitrary. Therefore, the second test of illusory control is to compare ratings of two conditions (low and high outcome density) that have the same contingency. Here, we carried out both tests simultaneously using the multifactorial analysis of variance (ANOVA) and follow-up tests.

On average, low BDI participants showed the illusion of control. They rated their control as 14.90 (SE 5.37), whereas high BDIs rated their control as nearer to zero (mean 1.16, SE 5.98). This main effect of BDI group was significant and confirmed that low BDI groups produced reliably higher ratings of control than participants with higher levels of depression: *F*_1,91_=4.05, mean squared error (MSE) 6671.40, *P*=.047, partial eta square=0.04, 90% CL 0.0004-0.1271. Subsequent single samples *t* tests comparing ratings to a criterion accuracy value of 0 showed that, for low BDI participants, 4 out of 5 action ratings were significantly higher than 0, all *t*s>2, all *P*s<.05, evidencing illusory control. For more dysphoric, high BDI participants, 5 out of 5 action ratings were not reliably different to 0, all *t*s<1.16, and all *P*s>.25. However, the BDI by outcome density interaction was not reliable (*F*_1,91_=0.63, MSE 6671.40, *P*=.43, partial eta square=0.0007), showing that the low BDI trend towards larger absolute ratings was not only evident in high outcome density conditions. This shows that non-depressed participants tended toward perceiving that they had more control over the occurrence of the auditory stimulus than depressed participants did whose ratings represented lower levels of perceived control.

### Alternative Cause Intervention Effect

**Figure 3:**
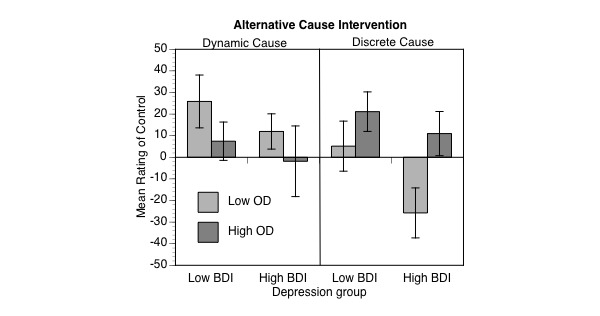
Mean ratings of control as a function of outcome density, Beck Depression Inventory (BDI) group and alternative cause intervention (error bars correspond to standard errors of the mean).

Figure 3 suggests that, as we predicted, instructions on the nature of the alternative cause enhanced perceptions of control. Only when participants were instructed that the alternative cause was a discrete entity did they show evidence of the healthy illusory control.

The analysis described above supported this observation because the interaction between instructions and outcome density was reliable: *F*_1,91_=7.05, MSE 6671.40, *P*=.009, partial eta square=.07, 90% CL 0.01-0.1674. Follow-up simple effects analyses confirmed this pattern and showed that the high outcome density conditions received higher ratings than low outcome density conditions only with discrete cause instructions (*F*_1,91_=6.06, MSE 1334.28, *P*=.02, partial eta square=.062, 90% CL 0.0064-0.1548) and not with dynamic cause instructions (*F*_1,91_=1.85, MSE 1334.28, *P*=.18, partial eta square=.02, 90% CL 0-0.0887).

These findings show that perceived control can be increased by providing people with simple instructions about the nature of alternative causes. Participants who compared their own control to discrete alternative causes produced higher control ratings.

## Discussion

### Principal Considerations

We used a mobile phone app in order to embed and measure perceived control occurring in participants’ everyday lives as naturalistic mobile phone interactions. The app was designed such that participants’ actions had no control over the occurrence of an auditory stimulus. Participants without signs of depression overestimated their control, whereas participants showing mild levels of depression rated their control as close to zero. This study provides the first objective demonstration of illusory control and depressive realism in a real-world setting.

Until now, the basic experimental findings of illusory control have never been rigorously tested outside the laboratory. This has raised important methodological and theoretical concerns, in particular around external and ecological validity, of generalizability from one very specific control situation to the whole of life [17] and of the difference between behavior instructed in the laboratory and that occurring naturally in the real world [18]. Such basic methodological critiques of perceived control research are well acknowledged in relation to depression [19] but not really discussed in the general health literature in which the concept of control is so frequently used. This gap has limited how our theoretical understanding of illusory control can be extrapolated to develop interventions, such as that described and tested here.

Notably, the instruction intervention significantly influenced all participants’ ratings of control. As we predicted, when the alternative cause was described as a discrete, constantly present entity (the mobile phone network), people judged that they had more control when the auditory stimulus occurred frequently (eg, [15]). Conversely, when people were informed that alternative causes were dynamic (ie, place) and would change throughout the experience, there was no evidence of illusory control effect. This finding suggests that the intervention used in this study, which was based on theoretical accounts of how people use alternative causes to evaluate their own control [14,21] was effective and successful.

This finding is consistent with work that shows that the perceived strength [28], salience [35], or exposure [16] of alternative causes, such as the context or other causes, competes with the cause under consideration for perceived control [15]. However, several things are important to note for those interested in designing perceived control interventions because the direction of the change in perceived control will depend on a number of key factors. First, the degree of control actually present in a situation is critical. When the person does have control, a strong alternative cause will reduce perceived control [28]. Second, the effect of any intervention or experimental manipulation, which changes the salience of or exposure to the alternative cause, will depend on what the person learns about the alternative cause as a result. So, for example, Vadillo et al [35] introduced a “difficult to ignore” alternative cause into their laboratory procedure, which seemed more strongly related to the outcome than participants’ actions. Msetfi et al [16] introduced a long delay into the procedure during the time that the alternative cause was present. This allowed participants to learn that the alternative cause was only weakly related to the outcome, which boosted their own perception of control. Both of these findings show how circumstances and information, which are present, can differentially influence people’s perceptions of their own control in very similar “no control” situations. This is critical information for those working in the health space, as perception of control is a very important mediator of health outcomes [4], which can be influenced unintentionally, as well as through intervention.

### Strengths and Limitations

It is important to consider whether the experimental procedure was a valid test of the contingencies that we planned for participants to experience. This is because it has been acknowledged that changes in participant behavior can actually affect the contingencies they are exposed to [18,24,36]. Our concern here was missed trials. However, careful scrutiny of the data recorded on each trial showed that even though participants missed some trials, they experienced the contingencies as programmed. This alleviates an important concern.

Another limitation is that our implementation of the contingency judgment task was an extremely crude analogue of real-life user–mobile phone interactions. However, in this first test of illusory control and depressive realism outside the laboratory, it was important to implement a real-life contingency task that was as similar as possible to lab procedures in order to provide the replication required. Our future studies will not only be able to provide a more sophisticated and naturalistic interface but will also collect richer data, including concurrent natural activities and behaviors, as well as mood and well-being ratings over longer periods of time. We also note that we have used similar nonclinical BDI criteria to test for depressive realism as the majority of the key studies in the literature [8,16]. This means that the generalizability of our findings to clinical depression is questionable, and we make no strong claims in this regard other than to state that we have replicated other depressive realism findings as tested elsewhere in the real world.

While we acknowledge the limitations of our study, especially in relation to the relatively controlled nature of the user-phone interactions we designed, a theoretically important aspect of ecological validity has been introduced into the procedure. This is the nature of the context or the alternative cause, which is fundamentally different in the laboratory to the dynamic, constantly changing contexts of real-world control situations. To date, most human learning studies simulate context using discrete cues [37] or places represented by pictures displayed on a computer screen [38] while a few have used an actual place [39]. It has been assumed that all such contexts “work” the same way in learning, and current theoretical models do not differentiate between them (see [40] for an extended discussion on context and learning). Our findings here suggest, to the contrary, that the representational content of context can change patterns of learning. This further emphasizes the importance of translating experimental research into the real world in order to fully use our theoretical knowledge to develop interventions.

### Conclusion

The findings of this study show convincingly that when perceptions of control are measured in relation to an objective standard, biased estimates of control do correlate with mental healthiness, with illusory control being the healthiest type of control (eg, [11,19,36]). Importantly, the simple theoretically motivated intervention, which was designed to influence people’s ability to learn about the power of the alternative cause in contrast to their own control, was effective in increasing people’s perceptions of being “in control.” Finally, this study further demonstrates the power of mobile phone technologies for use in experimental and intervention research. This not only provides the opportunity to test psychological theory in novel ways embedded in a person’s everyday environment but also to collect ever richer data about natural behaviors.

## Appendix

Multimedia Appendix 1 Full instructions.

Multimedia Appendix 2 Analysis of variance (ANOVA) table.

## Abbreviations

BDI: Beck Depression Inventory
CL: confidence limits
DASS: Depression Anxiety Stress Scales
MSE: mean squared error
