# Supplementary material for: Using Mobile Phones to Examine and Enhance Perceptions of Control in Mildly Depressed and Nondepressed Volunteers: Intervention Study
Source: JMIR Mhealth Uhealth. 2018 Nov 9;6(11):e10114. doi: 10.2196/10114 (PMC6251979; doi:10.2196/10114)
Supplement: Multimedia Appendix 1 [file mhealth_v6i11e10114_app1.pdf]

Multimedia Appendix 1. Full instructions

Instructions from the mobile phone app: Discrete context version *(highlighting shows differences with dynamic context version*)

Instructions 1

In this study, you will be asked to interact with your phone over the course of the day. During the day, every so often, an alert will sound (like when you receive a text message).
Click on the alert button below to see what this will look like:

Instructions 2

When you hear the notification, you should access the notification and click on it. This will open a new screen which has a blue button on it.

This is your opportunity to test whether your pressing the blue button controls the boing sound. You will have lots of opportunities to press the blue button over the course of the day and will receive lots of alerts.

Instructions 3

In order to test whether you can control the sound by pressing the blue button, you should press the blue button on roughly half of the alerts.
On other alerts, you should leave the blue button, not press it, and see what happens.
This does not mean that you should not respond to the alert, just that you may choose not to press the blue button on roughly half of the opportunities.

Instructions 4

Several times throughout the day we will ask you to rate the control you have over the boing sound. You will make your rating using a number wheel like the one shown below.

- Values near to +100 end indicates you feel you have total control.
- Values near to -100 end indicate you feel your actions prevent the boing sound.
- Values near to Zero, means you feel you have no control over the sound.

You can put the wheel anywhere in between these values to show your feelings.

Instructions 5

We will also ask you to rate how much control external factors have over the occurrence of the boing sound. This could be factors related to the phone, the mobile network or anything apart from your actions.

You will make your rating using a number wheel like the one shown below.

- Values near to +100 end indicates you feel external forces have total control.
- Values near to -100 end indicate you feel external forces prevent the boing sound.
- Values near to Zero, means you feel external forces have no control over the sound.

You can put the wheel anywhere in between these values to show your feelings.

Instructions 6

Now you have read all the instructions, please click on the start button below to start the study.

Instructions from the mobile phone app: Dynamic context version *(highlighting shows differences with discrete context version)*

Instructions 1

In this study, you will be asked to interact with your phone over the course of the day. During the day, every so often, an alert will sound (like when you receive a text message).
Click on the alert button below to see what this will look like:

Instructions 2

When you hear the notification, you should access the notification and click on it. This will open a new screen which has a blue button on it.

This is your opportunity to test whether your pressing the blue button controls the boing sound. You will have lots of opportunities to press the blue button over the course of the day and will receive lots of alerts.

Instructions 3

In order to test whether you can control the sound by pressing the blue button, you should press the blue button on roughly half of the alerts.
On other alerts, you should leave the blue button, not press it, and see what happens.
This does not mean that you should not respond to the alert, just that you may choose not to press the blue button on roughly half of the opportunities.

Instructions 4

Several times throughout the day we will ask you to rate the control you have over the boing sound. You will make your rating using a number wheel like the one shown below.

- Values near to +100 end indicates you feel you have total control.
- Values near to -100 end indicate you feel your actions prevent the boing sound.
- Values near to Zero, means you feel you have no control over the sound.

You can put the wheel anywhere in between these values to show your feelings.

Instructions 5

We will also ask you to rate how much control the place you are located in has over the occurrence of the boing sound. It's important to note that you will change your location throughout the day. The place you are in could affect whether the boing sound occurs, regardless of your actions.

You will make your rating using a number wheel like the one shown below.

- Values near to +100 end indicates you feel external forces have total control.
- Values near to -100 end indicate you feel external forces prevent the boing sound.
- Values near to Zero, means you feel external forces have no control over the sound.

You can put the wheel anywhere in between these values to show your feelings.

Instructions 6

Now you have read all the instructions, please click on the start button below to start the study.
