# Supplementary material for: Using Mobile Phones to Examine and Enhance Perceptions of Control in Mildly Depressed and Nondepressed Volunteers: Intervention Study
Source: JMIR Mhealth Uhealth. 2018 Nov 9;6(11):e10114. doi: 10.2196/10114 (PMC6251979; doi:10.2196/10114)
Supplement: Multimedia Appendix 2 [file mhealth_v6i11e10114_app2.pdf]

Multimedia Appendix 2. ANOVA

Table 1. *ANOVA table for analysis of ratings of action control*

Within subjects effects	F	p	η_p^2
Block	.441	.779	.005
Block * DP-exp	.523	.719	.006
Block *OD	.273	.895	.003
Block * BDI	.453	.770	.005
Block * Context	.395	.812	.004
Block * OD * BDI	.566	.687	.006
Block * OD * Context	1.746	.139	.019
Bblock * BDI * Context	.273	.895	.003
Block * OD * BDI * Context	.813	.517	.009
Between subjects effects			
DP-exp	0.732	0.394	0.008
OD	0.413	0.522	0.005
**BDI**	**4.053**	**0.047**	**0.043**
Context	1	0.32	0.011
OD * BDI	0.625	0.431	0.007
**OD * Context**	**7.046**	**0.009**	**0.072**
BDI * Context	0.314	0.577	0.003
OD * BDI * Context	0.257	0.613	0.003

NB: $df_{within} = 4, 364$; $df_{between} = 1, 91$; $MSE_{within} = 1651.30$; $MSE_{between} = 6671.40$
